# Integrating multi-atlas neuroimaging data for robust biomarker identification in neuropsychiatric disorders

**DOI:** 10.3389/fpsyt.2026.1723214

**Published:** 2026-03-19

**Authors:** Lijun Zhou, Yunfei Liu, Kaiyuan Wang, Xiaoyan Wang, Meirong He, Hongru Zhu, Junran Zhang

**Affiliations:** 1 School of Electrical Engineering, Sichuan University, Chengdu, China; 2 School of Electrical Engineering, Northwest Minzu University, Lanzhou, China; 3 Mental Health Center, National Center for Mental Disorders, West China Hospital, Sichuan University, Chengdu, China; 4 Med-X Center for Informatics, Sichuan University, Chengdu, China

**Keywords:** autism spectrum disorder, post-traumatic stress disorder, functional connectivity, multi-atlas, deep learning, graph neural network

## Abstract

**Introduction:**

Accurate diagnosis of neuropsychiatric disorders like autism spectrum disorder (ASD) and post-traumatic stress disorder (PTSD) is challenging due to complex disruptions in brain functional connectivity. Existing graph neural network (GNN) methods are often limited by reliance on single-atlas representations.

**Methods:**

We propose MSAT-LAFFNet, a multi-atlas GNN framework integrating a Structure-Aware Graph Transformer (SAT) for enhanced structural encoding and a Lightweight Attentional Feature Fusion network (LAFFNet) for adaptive cross-atlas fusion. The model was validated on the public ABIDE-I dataset and a private PTSD dataset (n=138).

**Results:**

Our model achieved an AUC of 82.9%/accuracy of 81.59% on ABIDE-I (ASD) and an AUC of 89.96%/accuracy of 89.45% on the PTSD dataset, outperforming competing methods. Interpretability analysis identified disorder-specific overlapping abnormal regions, including the right middle frontal gyrus in ASD and the left amygdala in PTSD, linking them to known network dysfunctions.

**Conclusion:**

MSAT-LAFFNet demonstrates superior classification performance and provides neurobiologically interpretable findings, showing potential as an effective tool for the auxiliary diagnosis of disorders characterized by disrupted functional networks.

## 1 Introduction

A core neuropathological hallmark of neuropsychiatric disorders such as autism spectrum disorder (ASD) and post-traumatic stress disorder (PTSD) is the dysregulation of neural connectivity networks (1–5). Whether such network abnormalities can be quantitatively characterized and reliably evaluated using neuroimaging tools, and further serve as diagnostic biomarkers, has become a central research focus. ASD is an early-onset neurodevelopmental disorder marked by persistent deficits in social communication and interaction, accompanied by restricted and repetitive behaviors, interests, or activities (1, 2). In contrast, PTSD is typically triggered by exposure to severe traumatic events such as warfare, natural disasters, or serious accidents, and is clinically characterized by intrusive memories, hypervigilance, avoidance of trauma-related cues, and negative alterations in mood and cognition (4, 5). Despite their distinct etiologies and symptom profiles, neuroimaging studies (6, 7) have revealed that both disorders share functional connectivity (FC) abnormalities within frontal-temporal circuits and limbic networks. However, how to precisely characterize these FC abnormalities, and whether they can serve as reliable diagnostic biomarkers, remains an open question (8).

Resting-state functional connectivity networks (FCNs) are increasingly regarded as promising neuroimaging biomarkers for neuropsychiatric disorders, including ASD and PTSD (5, 9). In ASD, disrupted FC typically manifests as long-range prefrontal-temporal hypoconnectivity co-occurring with local hyperconnectivity (1). PTSD, by contrast, is associated with decoupling between the default mode network (DMN) and limbic regions (4). FCN analysis, which defines neuroanatomical regions of interest (ROIs) based on brain atlases and quantifies connectivity via temporal correlations, offers a pathway to explore these pathological mechanisms (10). Machine learning and deep learning approaches applied to FCNs have achieved moderate success; for example, support vector machines (SVM) reached 68.7% accuracy in classifying ASD subtypes from healthy controls (HCs) (11), while autoencoder-based models attained 75% accuracy in cross-site PTSD classification (12). Yet, these methods rely on vectorizing the symmetric FC matrix into one-dimensional features, which discards graph-theoretic properties and neuroanatomically constrained topology (13, 14). This limitation presents a significant drawback for investigating dysregulation within neural connectivity networks underlying neuropsychiatric disorders such as ASD and PTSD. This loss of structural information diminishes the ability to detect subtle, disease-relevant connectivity abnormalities, such as disruptions in the prefrontal-amygdala inhibitory pathway in PTSD. Consequently, the capacity of conventional models to capture clinically meaningful brain network dysregulation remains limited.

Given the limitations of conventional machine learning and deep learning methods in classifying disorders using FCNs, graph neural networks (GNNs) have emerged as a promising alternative due to their ability to process non-Euclidean graph-structured data. Unlike traditional vectorization-based approaches, GNNs preserve the topology of FCNs by iteratively aggregating neighborhood information that respects anatomical constraints (15). Empirical evidence shows that GNNs can improve classification accuracy by approximately 9–18% over conventional methods (16–23). For example, BrainGNN integrates topological and functional features derived from fMRI, achieving 83.4% accuracy in ASD classification while localizing dysregulated brain regions (16). Similarly, Zhang et al. achieved 82.05% accuracy in ASD classification using graph contrastive learning with dual topological augmentation (19). In the same year, Huynh et al. proposed a GAN-based data augmentation framework combined with GCN, attaining 75.16% accuracy in ASD classification and 95.56% on a private PTSD dataset (23). Despite these advances, existing GNN models face notable challenges in neuropsychiatric disorder analysis. First, limited network depth reduces their ability to capture complex patterns of functional brain connectivity, particularly those involving interactions across frontal and temporal regions (24). Although recent GNN-Transformer hybrids aim to capture complex brain connectivity patterns and interactions between distant brain regions (25, 26), they still lack the ability to detect subtle abnormalities in local network structures. Second, current GNN frameworks typically rely on a single atlas, which restricts feature characterization and lacks multi-scale feature extraction capacity. This limitation hinders the accurate modelling of the complexity and inter-individual variability of FCNs (27, 28). To reduce such biases, multi-atlas strategies have been introduced for neuroimaging feature extraction (18, 29–31). For instance, Han et al. (18) proposed a direct concatenation scheme for multi-atlas fusion in Alzheimer’s disease diagnosis, which substantially improved diagnostic performance. A similar splicing strategy is implemented in recent literature (31). However, direct merging approaches often discard fine-grained anatomical constraints and overlook functional disparities among disease-implicated ROIs (32, 33). More critically, they fail to establish mechanistic links between predictive features, dysregulated regions, and disorder-specific connectivity patterns, thereby limiting clinical interpretability. Meanwhile, recent research trends also favor exploring more efficient cross-graph attention fusion mechanisms, integrating causal reasoning to improve model interpretability and generalization capabilities, and designing lightweight architectures to enable clinical deployment (34–36).

To overcome these limitations, we propose an improved GNN-based classification framework called the multi-atlas structure-aware graph Transformer with a lightweight attentional feature fusion network (MSAT-LAFFNet). This framework integrates multi-scale neuroanatomical priors with advanced graph learning to achieve accurate classification of neuropsychiatric disorders and precise characterization of aberrant FCNs.

The primary contributions of this work are summarized as follows:

- MSAT-LAFFNet, a novel GNN-based multi-atlas framework that seamlessly incorporates neuroanatomical priors into the analysis of resting-state FCNs is proposed.
- The structure-aware graph Transformer (SAT) was designed, which leverages structural similarity in its attention mechanism to jointly capture local and global connectivity features, enhancing the representation of disease-specific abnormalities.
- The lightweight attentional feature fusion network (LAFFNet) was developed, an attentional fusion module that adaptively quantifies the contribution of distinct atlases through learnable weights, improving both classification performance.
- The proposed method was developed, which enhances clinical interpretability through the identification of disorder-specific patterns in overlapping abnormal brain regions and disrupted functional connectivity.

## 2 Materials and methods

### 2.1 Datasets and preprocessing

In the article, we used two clinically challenging neuropsychiatric disorder datasets: the publicly available ABIDE-I dataset (37) and a proprietary PTSD dataset. For each dataset, we describe the cohort composition and the preprocessing pipeline.

ABIDE-I is a heterogeneous, multi-center dataset. After quality inspection, 403 individuals with ASD and 468 normal controls (NCs) were included in our analysis. Detailed demographic and clinical information is provided in **Table 1**. To ensure reproducibility and enable fair comparisons with prior work, we used the preprocessed connectomes project pipeline (38). The preprocessing steps were as follows: (i) slice-timing correction; (ii) motion correction; (iii) global mean intensity normalization; (iv) regression of nuisance signals (head motion, respiration, cardiac activity, and scanner drift; (v) band-pass filtering (0.01–0.1 Hz); (vi) functional image alignment; (vii) registration to standard space.

**Table 1:** Demographic information of the 871 subjects in the ABIDE-I dataset.

Subject information	ASD (403)	NC (468)	*t*-test (*t* value)	*t*-test (*p* value)
Age (year)	17.07 ± 7.95	16.85 ± 7.24	0.4360	0.6629
Sex (Male/Female)	349/54	378/90	–	–
ADOS (total)	11.17 ± 3.11	1.33 ± 1.12	12.5598	<0.001

The PTSD dataset comprised a clinically challenging cohort of 138 earthquake survivors from West China Hospital of Sichuan University. After rigorous quality control, 59 PTSD patients and 51 trauma-exposed non-PTSD controls were retained for analysis. Demographic and clinical characteristics are summarized in **Table 2**. Preprocessing was performed using the graph theoretical network analysis (GRETNA) toolbox (39). The steps included: (i) discarding the first 10 time points (resulting in 195 time points for analysis); (ii) slice-timing correction and realignment to reduce head motion artifacts; (iii) regression of nuisance signals (head motion, respiration, cardiac activity, and scanner drift); (iv) band-pass filtering (0.01–0.08 Hz).

**Table 2:** Demographic information of the 110 subjects in the PTSD dataset.

Subject information	PTSD (59)	Non-PTSD (51)	*t*-test (*t* value)	*t*-test (*p* value)
Age (year)	44.98 ± 7.39	46.24 ± 6.46	0.9516	0.3434
Sex (Male/Female)	16/43	16/35	–	–
Education (year)	8.63 ± 2.62	7.29 ± 3.81	-2.1138	0.0866
CAPS (total)	69.34 ± 21.01	10.51 ± 8.84	18.6125	<0.001

The ABIDE-I data consistent with most studies using this public dataset (26, 29). For the PTSD dataset, parameters such as the band-pass filter were optimized in line with previous processing standards for this batch of data at West China Hospital (40), in order to ensure signal quality and internal consistency. All analyses were performed on high-quality preprocessed signals. Crucially, our method focuses on the topology of functional connectivity, which remains robust to reasonable variations in preprocessing parameters.

### 2.2 MSAT-LAFFNet

**Figure 1:**
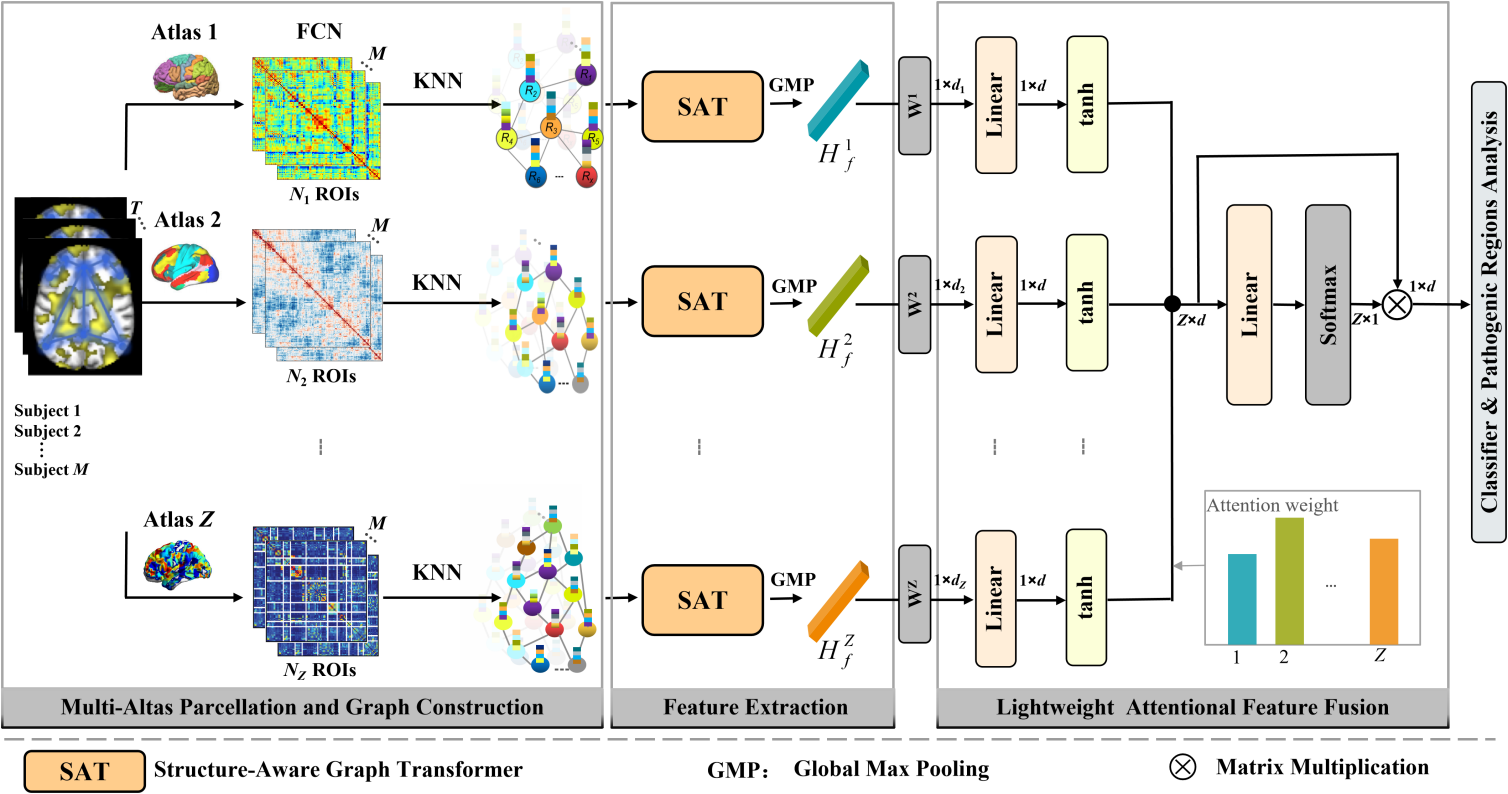
Overview of the proposed method.

The proposed diagnostic classification framework MSAT-LAFFNet consists of three integrated components: (i) multi-atlas parcellation and graph construction, (ii) structure-aware graph feature extraction, and (iii) multi-graph lightweight attention fusion with classification. As illustrated in **Figure 1**, the workflow proceeds in three stages:

- Multi-atlas parcellation and graph construction. Multiple prior atlases are employed to derive FC matrices from the preprocessed signals. Graphs are then constructed by sparsifying edges using the K-nearest neighbors (KNN) algorithm. In this study, we set K = 15. This value was chosen to generate a sparsely connected graph, and during experiments, the model demonstrated stable performance around this parameter. For further details of graph construction, see **Supplementary Material 1.1**.
- Structure-aware feature extraction. Graphs at different spatial scales are processed by the SAT module, which captures both fine-grained local structural details and long-range node interaction patterns.
- Feature fusion and classification. LAFFNet adaptively integrates multi-scale features through learnable weighting coefficients, and the fused representation is passed to the classifier. This design enables both accurate diagnostic recognition and clinically meaningful interpretability.

Atlas Description. A neuroanatomical atlas defines functionally coherent brain regions by integrating structural and connectivity properties (41). The automated anatomical labelling (AAL) atlas (42), for example, partitions each cerebral hemisphere into 39 cortical and 6 subcortical regions based on anatomical landmarks, and remains one of the most widely adopted neuroimaging references. Connectivity-driven approaches, in contrast, generate fine-grained subdivisions that capture inter-regional interactions and provide insights into neural organization principles (43, 44). Representative examples include the Craddock 200 (CC200) atlas (45), which defines 200 functionally homogeneous regions through multi-sample clustering analysis, and the Brainnetome (BN) atlas (46), which delineates 246 regions (210 cortical and 36 subcortical) using connectional architecture and combined anatomical-functional information. Within each BN region, voxels exhibit consistent connectivity profiles, reflecting coherent functional organization. Collectively, these atlases span coarse-to-fine resolutions and offer complementary strengths. It is worth noting that additional atlases can also be seamlessly incorporated into our framework.

**Figure 2:**
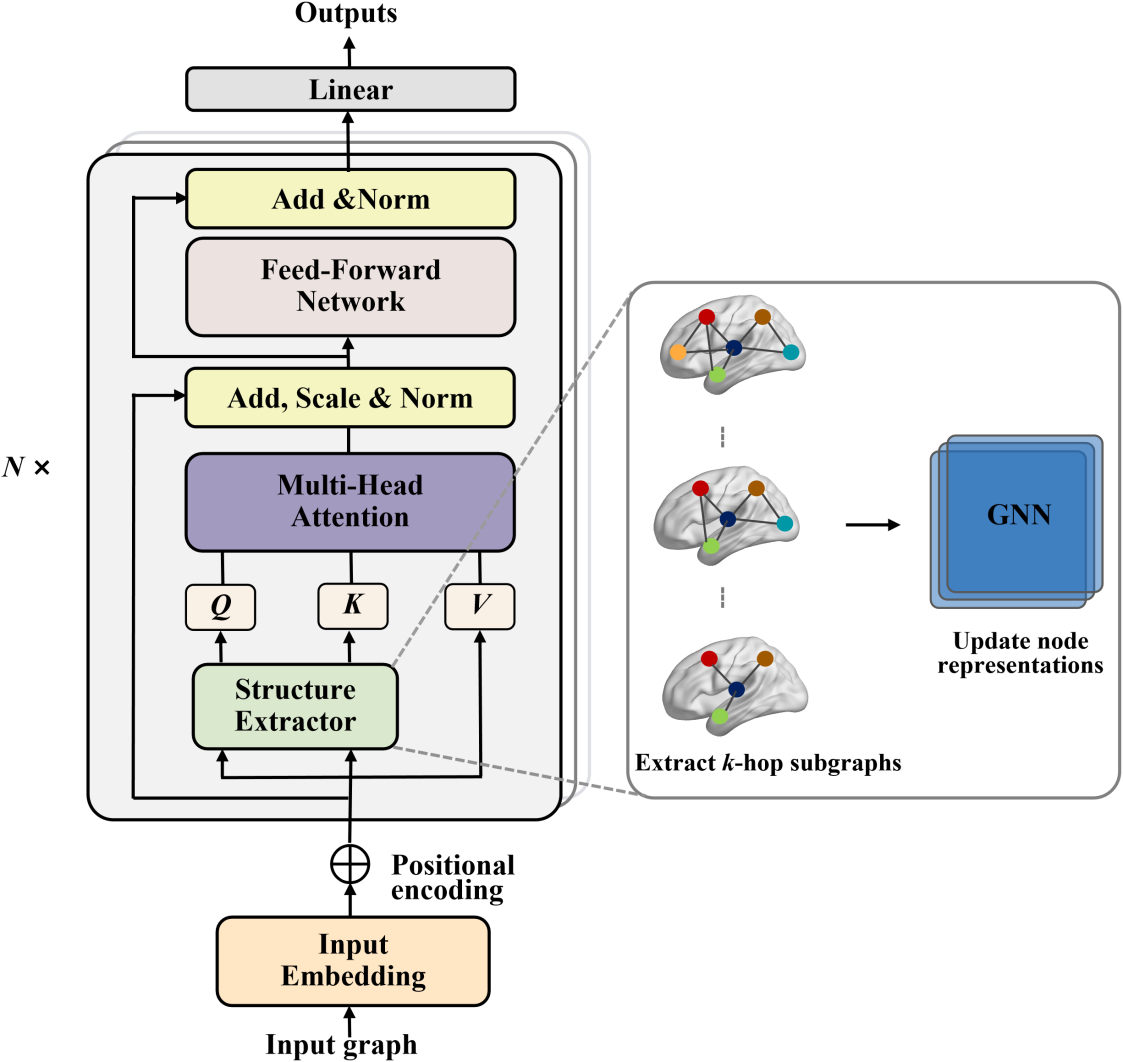
Overview of the SAT layer that uses the *k*-subgraph GNN extractor.

Structure-aware feature extraction. To obtain the comprehensive feature representation from different atlases for brain disorder diagnosis, we design a SAT module that extracts ROI features from each atlas separately. The flexible SAT mechanism that encodes topology via *k*-hop subgraph embeddings, thereby capturing both pairwise ROI interactions and higher-order structural dependencies (**Figure 2**). For further details and formula derivation, please refer to **Supplementary Material 1.2**.

Feature fusion and classification. Early multi-atlas studies on disease classification (27, 28), including our prior work (47), often adopted direct feature concatenation across atlases. While straightforward, this strategy neglects the varying contributions of different features, potentially introducing noise and irrelevant information. In fMRI data, features extracted from distinct atlases differ markedly in semantic level (*e.g*., local vs. global patterns). Simply concatenating these features fails to capture their relative importance and inter-atlas correlations and may even impair classification performance (48). Attention mechanisms, particularly multi-head self-attention (MHSA) (49), have demonstrated strong capability in modelling feature dependencies and are natural candidates for multi-graph feature fusion. However, conventional MHSA exhibits two key limitations in this context: (i) the softmax-based attention computation tends to overemphasize features with high correlations even when they are weakly discriminative, and (ii) the quadratic computational cost O(N²) for pairwise feature interactions hinders scalability in multi-atlas settings. To address these issues, we propose LAFFNet, inspired by the lightweight attention block for cross-modal text-to-video matching in reference (48). Instead of relying on Q-K-V correlation modelling, LAFFNet directly learns feature importance weights through a parameter-efficient mechanism. The model’s final output is achieved using a classifier for disease diagnosis. For specific implementation details of LAFFNet and classifier, see **Supplementary Material 1.3**. In summary, LAFFNet provides an efficient and interpretable solution for multi-atlas feature fusion, outperforming both direct concatenation and MHSA in terms of scalability, classification accuracy, and interpretability.

## 3 Experimental and results

### 3.1 Implementational details

All experiments were conducted on a shared GPU cluster equipped with NVIDIA GTX 3090 cards running in parallel. The proposed model was implemented using PyTorch and PyG. It consists of three graph convolutional layers and is trained with a batch size of 8. To mitigate overfitting, ReLU activation and dropout (set to 0.3) were applied. The learning rate was initialized at 0.001 and dynamically adjusted using a cosine annealing schedule. Optimization was performed with the AdamW optimizer, using cross-entropy loss with label smoothing as the training objective. Model hyperparameters were tuned through grid search, and training was carried out for 200 epochs.

To ensure robust and reliable evaluation, we adopted a nested n-fold cross-validation strategy. The diagnostic performance of the model was assessed using four metrics: accuracy (ACC), sensitivity (SEN), specificity (SPE), and the area under the receiver operating characteristic curve (AUC). Detailed calculation procedures are provided in literature (36).

### 3.2 Classification performance

The performance of the proposed MSAT-LAFFNet framework was comprehensively evaluated through rigorous comparative experiments on two independent datasets. For benchmark comparisons, multiple state-of-the-art classification methods were implemented under identical experimental protocols to ensure fairness. The competing eight methods included: SVM (11), BrainnetCNN (50), GCN (21), BrainGNN (16), MCRLN (51), MGCA-RAFFNet (30), SGCN (52), and MHAHGEL (29). They all adopt the exact same nested cross-validation framework as our proposed model. Specifically, inner-loop cross-validation is employed on the outer-loop training set of each fold for hyperparameter search and selection, ensuring that the chosen parameters generalize to the validation set of that fold. Performance is ultimately reported on an independent test set. All comparison models underwent systematic hyperparameter tuning. The optimal hyperparameter settings for the comparison models are detailed in **Supplementary Material 2.1**.

To ensure fairness, all comparative methods were evaluated using the same standardized datasets, with implementation codes obtained directly from the respective authors. All experiments were conducted under identical conditions as those used for our proposed framework. Evaluation protocols were adapted to dataset characteristics: the PTSD dataset (n=110) was evaluated with 5-fold cross-validation, while the larger ABIDE-I dataset (n=871) employed 10-fold cross-validation to account for its larger sample size (**Tables 3**, **4**). Selecting such cross-validation folds ensures that the requirements for both small- and large-sample validation are met, thereby guaranteeing the reliability and reproducibility of model evaluation (23, 30). On the ABIDE-I dataset (**Table 3**), MSAT-LAFFNet achieved superior results, with ACC = 81.59%, AUC = 82.9%, SEN = 80.26%, and SPE = 82.9%. On the PTSD dataset (**Table 4**), MSAT-LAFFNet consistently outperformed both traditional and recent methods, achieving improvements of at least 1.9% (ACC), 1.61% (SEN), and 2.54% (SPE) in PTSD vs. Non-PTSD classification. In terms of AUC, it also demonstrated gains of no less than 2.52% for PTSD vs. Non-PTSD. These comprehensive results confirm that MSAT-LAFFNet significantly outperforms existing methods across diverse datasets, highlighting its effectiveness and robustness for diagnostic classification tasks.

**Table 3:** Comparison of evaluation metrics across different methods for ABIDE-I dataset (%).

Method	Year	NC VS. ASD			
		ACC(%)	SEN(%)	SPE(%)	AUC(%)
SVM	–	63.19 ± 1.06	61.53 ± 1.21	58.21 ± 1.33	66.24 ± 0.54
GCN	2017	68.53 ± 1.32	67.58 ± 1.47	70.12 ± 1.26	71.72 ± 0.82
BrainnetCNN	2017	67.81 ± 1.27	66.35 ± 0.86	69.51 ± 0.77	74.19 ± 1.41
BrainGNN	2021	69.88 ± 2.33	66.73 ± 1.09	69.11 ± 1.45	72.46 ± 1.25
MCRLN	2023	72.24 ± 1.53	70.58 ± 1.94	71.17 ± 1.17	73.76 ± 1.28
MGCA-RAFFNet	2023	75.14 ± 1.77	76.08 ± 1.91	74.13 ± 1.17	76.33 ± 0.77
SGCN	2024	72.11 ± 1.41	67.39 ± 1.72	71.46 ± 0.59	73.65 ± 0.65
MHAHGEL	2024	78.29 ± 0.46	74.27 ± 1.51	77.03 ± 2.24	78.35 ± 0.47
**MSAT-LAFFNet (ours)**	**2025**	**81.08 ± 0.51**	**79.23 ± 1.03**	**80.31 ± 0.42**	**82.23 ± 0.67**

Results are expressed as “mean ± standard deviation.” Bold fonts indicates the best performance.

**Table 4:** Comparison of evaluation metrics across different methods for PTSD dataset (%).

Method	Year	PTSD VS. Non-PTSD			
		ACC(%)	SEN(%)	SPE(%)	AUC(%)
SVM	–	67.81 ± 1.09	69.36 ± 1.35	67.87 ± 1.18	70.35 ± 0.65
GCN	2017	72.36 ± 1.28	73.18 ± 2.03	72.41 ± 1.36	74.29 ± 0.71
BrainnetCNN	2017	74.22 ± 0.71	70.39 ± 0.73	73.67 ± 0.64	75.16 ± 0.73
BrainGNN	2021	77.82 ± 2.04	75.36 ± 2.47	79.1 ± 1.58	78.67 ± 0.33
MCRLN	2023	81.95 ± 1.53	73.58 ± 2.51	83.12 ± 1.82	82.68 ± 1.31
MGCA-RAFFNet	2023	85.54 ± 1.07	83.80 ± 1.31	84.52 ± 1.25	89.12 ± 0.13
SGCN	2024	82.68 ± 1.29	80.54 ± 0.56	84.29 ± 1.28	84.68 ± 1.32
MHAHGEL	2024	86.54 ± 1.01	84.27 ± 0.55	82.08 ± 2.02	87.12 ± 0.32
**MSAT-LAFFNet (ours)**	**2025**	**88.26 ± 1.19**	**85.13 ± 0.75**	**84.62 ± 1.57**	**89.51 ± 0.45**

Results are expressed as “mean ± standard deviation.” Bold indicates the best performance.

We conducted rigorous ablation experiments on the ABIDE-I and PTSD datasets to validate the contribution of each constituent module in MSAT-LAFFNet. The experimental results confirm that each module plays a critical role in improving diagnostic performance for both ASD and PTSD. These results are presented in **Supplementary Material 2.2**. We provide an in-depth evaluation of the computational complexity, as well as the impact of the hyperparameters of the proposed model, in **Supplementary Material 2.3, 2.4**.

### 3.3 Clinically interpretable results

#### 3.3.1 Contributions of different atlases

**Figure 3:**
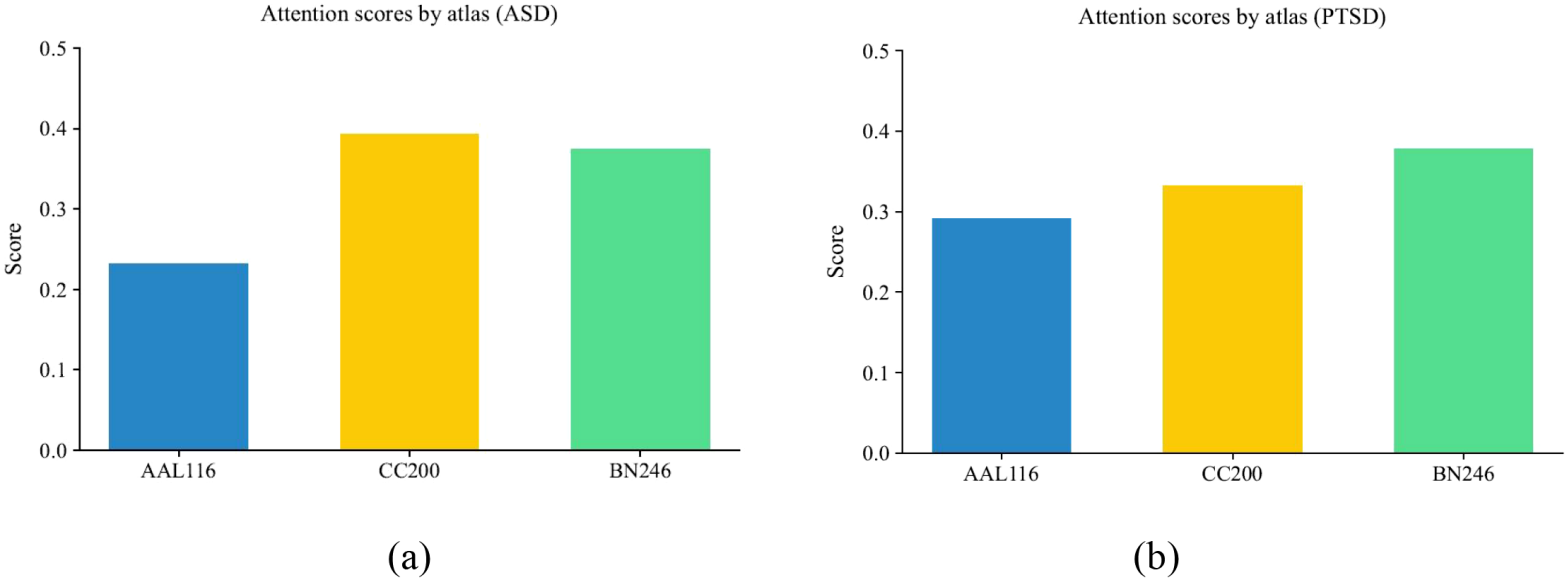
The atlas attention scores for the different classification tasks. **(a)** NC vs. ASD, **(b)** Non-PTSD vs. PTSD.

To better determine the suitability of different atlases for PTSD and ASD classification, we analyzed the attention weights from the LAFF module to derive atlas-specific attention scores within the optimal tri-atlas combination (AAL116+CC200+BN246), as shown in **Figure 3**. The results indicate that, for ASD classification, the CC200 atlas provides the highest attentional contribution (0.3932), whereas for PTSD classification, the BN246 atlas exhibits the largest contribution (0.3775). Notably, the atlas with the maximal classification contribution under tri-atlas fusion consistently aligns with the top-performing single-atlas configuration (CC200 for ABIDE-I; BN246 for PTSD), thereby providing empirical support for the neurobiological relevance of our selected multi-atlas strategy.

#### 3.3.2 Most discriminative brain regions

**Figure 4:**
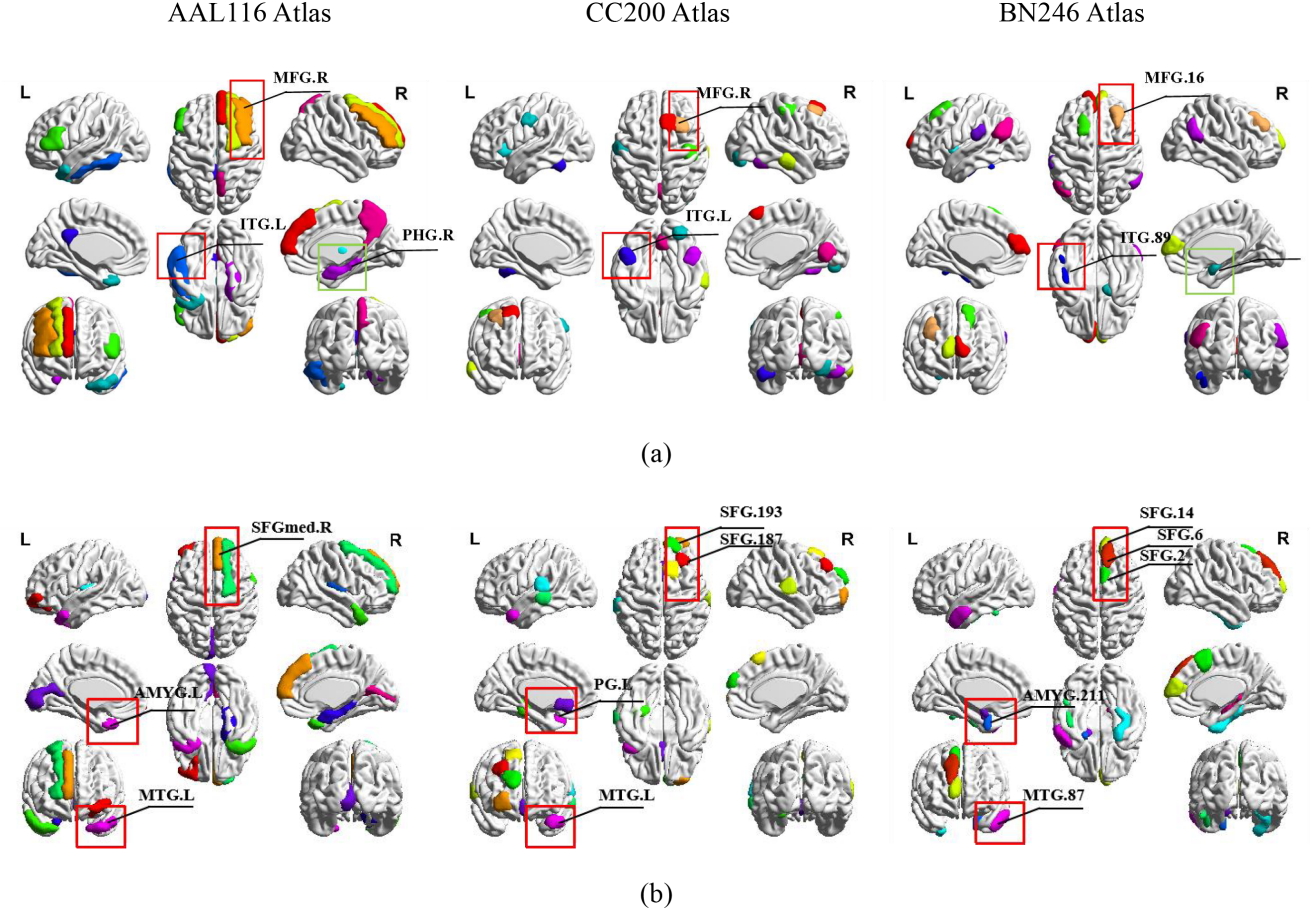
The brain regions with the top 10 attentional weights for different atlases and their overlapping regions across the three atlases. Red boxes indicate regions overlapping in all three atlases, while green boxes denote regions overlapping in two atlases (two-sample t-test, FDR-corrected p< 0.05). **(a)** NC vs. ASD, **(b)** Non-PTSD vs. PTSD.

The SAT module was leveraged to derive ROI-specific weights, facilitating the identification of potential neuroimaging biomarkers for ASD and PTSD. We list the 10 ROIs in each atlas with the highest discriminative regions across both datasets and their corresponding cortical regions in **Table 5**. Meanwhile, the top 10 most discriminative regions were mapped from each atlas (AAL116, CC200, BN246) using the BrainNet Viewer Toolbox (53), as shown in **Figure 4**. The top 10 ROIs were selected based on the average attention weights calculated by the model across all subjects in the training set. The attention weight is calculated using formula (4), which can be found in **Supplementary Material 1.2**. This aims to identify important neural features that are consistent across subjects in the model’s decision-making process.

**Table 5:** The top 10 discriminative ROIs and their corresponding cortical regions in the two datasets.

Cortical regions	ABIDE-I dataset			PTSD dataset		
	AAL116	CC200	BN246	AAL116	CC200	BN246
Frontal	SFGmed.R,MFG.R,SFGdor.R,IFGtriang.L	SFGmed.R,MFG.R,Insula.L	SFG.3,SFG.13,SFG,14,MFG.16	SFGmed.R,MFG.R,SFGdor.R	FP12.R,FP106.R,FP193.R,SFG.R	SFG.2,SFG.6,SFG,14
Temporal	MTG.L,ITG.L	MTG.L,ITG.L, MTGmed.R	ITG.89,PHG.114	MTG.L,TPomed.R,HES.L,HES.R	MTG.L,STG.L,PT.L,PT.R	MTG.87,ITG.89,FuG.104
Subcortex	THA.R	PoG.L	AMYG.212,INS.173	AMYG.L	PG.L	AMYG.211,AMYG.213,Hipp.218
Central	–	PrG.R	–		SC.L	–
Limbic	PHG.R	–	–	PHG.R	–	PHG.117
Parietal	PCUN.R,PCG.L	FuG.R,Calcarine.R	IPL.143,IPL.145	CAL.R	–	–

SFGmed.R – .the right medial superior frontal gyrus; MFG.R – the right middle frontal gyrus; SFGdor.R – the right superior frontal gyrus; IFGtriang.L – the left inferior frontal gyrus; MTG.L – the left middle temporal gyrus; ITG.L – the left inferior temporal gyrus; THA.R – the right thalamus; PHG.R – the right parahippocampal gyrus; PCUN.R – the right Precuneus; PCG.L – the left posterior cingulate gyrus; Insula.L – the left Insular gyrus; MTGmed.R – the right medial middle temporal gyrus; PoG.L – the left postcentral gyrus; PrG.R – the right Precentral gyrus; AMYG – amygdala; INS – Insular gyrus; IPL – Inferior parietal lobule; TPomed – Temporal pole: middle temporal gyrus; HES – Heschl gyrus; CAL.R – the right calcarine cortex; FP – frontal pole; STG.L – the left Superior temporal gyrus; PT – Planum temporale; SC – Subcallosal cortex; FuG – fusiform gyrus; Hipp – hippocampus.

#### 3.3.3 Most discriminative functional connectivity

**Figure 5:**
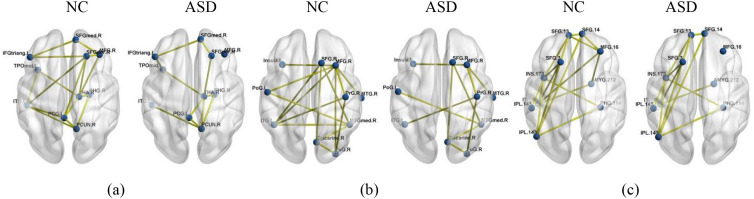
The functional connections among the top 10 discriminative ROIs for ASD classification tasks (two-sample t-test, FDR-corrected p< 0.05). **(a)** AAL116, **(b)** CC200, **(c)** BN246.

**Figure 6:**
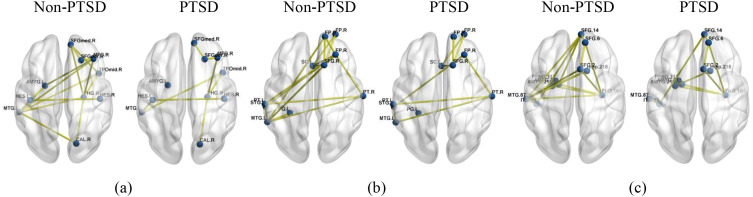
The functional connections among the top 10 discriminative ROIs for PTSD classification tasks (two-sample t-test, FDR-corrected p< 0.05). **(a)** AAL116, **(b)** CC200, **(c)** BN246.

To further explore the role of FC in these disease-related regions, we visualized the connectivity patterns among the top 10 most discriminative ROIs across atlases using the BrainNet Viewer Toolbox (53) (see **Figures 5**, **6**). The most discriminative functional connections were identified by evaluating the correlation between ROIs and sample labels using a significance test, with FDR-corrected p-values < 0.05 set as the significance threshold.

## 4 Discussion

This study proposes and validates an integrated framework that significantly enhances the classification performance for ASD and PTSD through the systematic integration of multi-atlas prior knowledge, structure-aware feature extraction, and adaptive feature fusion. The following discussion first examines the sources of its performance gain through ablation studies, followed by an in-depth analysis of its contribution to the clinical interpretability of ASD and PTSD.

Ablation studies were conducted to systematically evaluate the contribution of each core module to the overall performance. The fusion of the AAL116, CC200, and BN246 atlases yielded the best performance (**Supplementary Tables 2**, **3**), indicating optimal topological complementarity among their functional connectivity networks, a finding consistent with prior reports (29, 30). The LAFF module further enhanced inter-atlas fusion, outperforming our previous method (47), with the specific contributions of each atlas illustrated by the attention scores in **Figure 3**. However, adding more atlases degraded performance, likely due to increased feature redundancy and noise, aligning with observations in (35). Analysis of **Supplementary Figures 1**, **2** confirmed that the SAT module strengthens graph feature extraction, and its integration of subgraph extraction with a transformer boosts interpretability. Overall, the proposed model demonstrates robust performance while providing explicit insights into local network anomalies—a challenge for some recent GNN-Transformer hybrids (25, 26)—and maintains computational efficiency comparable to current state-of-the-art methods (34). This reliable performance foundation lends credibility to the neurobiological interpretations derived from the model.

Our interpretability analysis reveals key brain regions consistently identified across atlases, linking them to established neurobiological mechanisms of ASD and PTSD. As summarized in **Table 5**, frontal and temporal regions emerged as dominant neural substrates for both disorders, though with distinct emphases: in ASD, frontal areas play a primary role (54, 55), supported by temporal contributions, whereas in PTSD, temporal regions are foremost, followed by frontal involvement (56, 57). This pattern aligns with existing models of frontal-temporal network dysfunction in both conditions. More specifically, our method converged on overlapping abnormal regions that correspond closely to core symptom domains. In ASD (**Figure 4a**), the right middle frontal gyrus (MFG.R) and left inferior temporal gyrus (ITG.L) were consistently weighted across all atlases, along with the right parahippocampal gyrus (PHG.R) in two atlases. These regions are well-documented in ASD literature—MFG.R in working memory and social cognition, ITG.L in semantic processing, and PHG.R in episodic memory—directly relating to social, communicative, and repetitive behavioral symptoms (3, 11, 58). In PTSD (**Figure 4b**), the left middle temporal gyrus (MTG.L), right medial superior frontal gyrus (SFGmed.R), and left amygdala (AMYG.L) showed cross-atlas consistency. These areas are central to PTSD pathophysiology: MTG.L supports multimodal integration, SFGmed.R is a key node of the default mode network (DMN) involved in self-referential and emotional processing, and AMYG.L underlies threat detection and fear memory (40, 57, 59). Together, these region-level findings not only validate the ability of our framework to detect neurobiologically grounded, cross-atlas consensus features but also strengthen the link between the model’s discriminative features and the established frontal-temporal-limbic abnormalities reported in ASD and PTSD.

Building upon the identification of these key brain regions, we further examined the functional connectivity patterns among them. In ASD, the analysis of functional connectivity revealed a consistent pattern of weakened long-range connections, particularly between prefrontal and temporal regions. Key disruptions included reduced connectivity between the right middle frontal gyrus (MFG.R) and the left inferior temporal gyrus (ITG.L), as well as between MFG.R and the left amygdala (AMYG.L) and posterior cingulate gyrus (PCG.L) (**Figure 5**). These findings align with the “insufficient long-range connectivity” hypothesis in ASD (1, 58, 60, 61). Specifically, the decoupling of MFG.R from ITG.L may reflect impaired integration of social-perceptual information, while weakened MFG.R-AMYG connectivity could contribute to dysregulated emotional responses and social avoidance. Collectively, these results underscore that reduced prefrontal-temporal connectivity is a central network-level feature in ASD, likely underlying core difficulties in social information processing and contextual integration (30, 62).

Conversely, analysis of PTSD reveals a central mechanistic pattern: a pronounced decoupling between the DMN and the amygdala. As shown in **Figure 6**, patients with PTSD exhibited marked disruptions in long-range functional connectivity, most critically involving weakened links between key regions such as AMYG.L, the left middle temporal gyrus (MTG.L), and SFGmed.R. This DMN-amygdala decoupling is consistent with prior evidence linking these pathways to trauma-related pathology (4, 6, 63, 64). Mechanistically, the observed disconnect likely reflects a failure of top-down regulatory control from the DMN over limbic circuits, thereby compromising the integration of traumatic memories and the regulation of fear responses (65). This pattern stands in contrast to the weakened prefrontal-temporal connectivity characteristic of ASD, highlighting disorder-specific network signatures. Together, the connectivity profiles identified by the proposed model are consistent with established neurobiological models of PTSD and may contribute to the exploration of network-based biomarkers for differential diagnosis.

Based on the above analysis, the integrated framework developed in this study achieves high classification performance for ASD and PTSD and, crucially, establishes a interpretable link between diagnostic outcomes and underlying pathophysiology. By leveraging structure-aware attention and adaptive fusion mechanisms, the model identifies abnormal brain regions and functional connectivity patterns that are both disorder-specific and consistent across multiple atlases. These findings not only align with established pathological models but also point to specific network-level mechanisms of dysfunction. Consequently, this work advances beyond performance improvement to provide interpretations with potential clinical relevance.

## 5 Conclusion

This study introduces MSAT-LAFFNet, a multi-atlas, structure-aware graph Transformer framework with lightweight attentional feature fusion, for the diagnosis of ASD and PTSD using resting-state fMRI data. By incorporating structural similarity into the SAT module, the framework effectively overcomes limitations of conventional GNNs in encoding brain network topology. The LAFFNet module further enhances feature discriminability by adaptively integrating complementary cross-atlas information, capturing latent relationships across brain regions. Experimental evaluation on the ABIDE-I and private PTSD datasets demonstrates superior performance compared to existing methods, validated through extensive metrics, ablation studies, and cross-validation. Importantly, the framework provides interpretable insights into disease-specific neural mechanisms, identifying critical aberrant regions and connectivity patterns consistent with clinical findings, such as prefrontal-temporal deficits in ASD and DMN-amygdala decoupling in PTSD. The main limitations of this study are as follows: i) The influence of neuropsychiatric comorbidities (*e.g.*, ASD with ADHD, PTSD with depression) on connectivity patterns was not systematically addressed; ii) Potential effects of psychotropic medication on functional networks were not controlled, limiting generalizability to treatment-naive populations; and iii) Intra-diagnostic heterogeneity (*e.g.*, ASD subtypes, PTSD symptom clusters) suggests that subgroup-specific models may further enhance clinical utility. Overall, MSAT-LAFFNet establishes a robust, interpretable framework for auxiliary diagnosis of neuropsychiatric disorders and offers a practical pathway for integrating multi-atlas neuroimaging data into precision medicine applications.

## Data Availability

The original contributions presented in the study are included in the article/**Supplementary Material**. Further inquiries can be directed to the corresponding authors.
