# Supplementary material for: Integrating multi-atlas neuroimaging data for robust biomarker identification in neuropsychiatric disorders
Source: Front Psychiatry. 2026 Mar 19;17:1723214. doi: 10.3389/fpsyt.2026.1723214 (PMC13044150; doi:10.3389/fpsyt.2026.1723214)
Supplement: Supplementary file 1 [file SupplementaryFile1.pdf]

Supplementary Material

1 SUPPLEMENTARY METHODS

1.1 Multi-Atlas Parcellation and Graph Construction

Formally, suppose there are M subjects and Z atlases. For each subject, the Z atlases to parcellate brain space into $N_1, N_2, ..., N_Z$ ROIs, respectively. Each ROI time series has T time points, which is the same for all atlases. Then, let $G = (V, E, X)$ be an undirected graph, where nodes $V = \{v_i\}_{i=1}^{N}$ is the $N = \{N_1, N_2, ..., N_Z\}$ ROIs, and edge $E = \{e_{ij}\}_{N\times N}$ present the retained edges in the FC matrix (Pearson correlation coefficient values between ROI_i and ROI_j, $1 \leq i, j \leq N$) after sparsification according to the KNN scheme. Let $X = \{x_1, x_2, ..., x_N\} \in \mathbb{R}^{N\times N}$ be the FCN matrix (node feature matrix) of a training subject. When the Zth altas partition parcellates the brain into N_Z) ROIs, the resulting graph is denoted as $G_Z = (V_Z, E_Z, X_Z)$ and its node feature matrix X is expressed as follows:$X_Z = \{x_1, x_2, ..., x_{N_Z}\} \in \mathbb{R}^{N_Z\times N_Z}$.

1.2 Structure-Aware Graph Transformer Feature Extraction

Although numerous message-passing strategies have been developed, conventional GNNs still face fundamental limitations (1). Recently, Transformer-based architectures have become prominent in graph representation learning by replacing local neighborhood aggregation with global attention. These methods rely on explicit structural encodings, such as positional embeddings, to capture graph topology (2). However, most existing approaches primarily encode node positional relations while overlooking explicit structural equivalence. As a result, they fail to account for higher-order contexts such as isomorphic local substructures or functionally similar nodes in different neighborhoods (3). For instance, FCNs from a patient and a healthy control may produce identical shortest-path embeddings despite pathological differences, thereby losing critical discriminative information and increasing misclassification risk. By explicitly modelling multi-scale structural disparities, such as hub connectivity or modular organization, diagnostic accuracy can be significantly improved (4). To address this gap, we introduce a flexible structure-aware self-attention mechanism that encodes topology via k-hop subgraph embeddings, thereby capturing both pairwise ROI interactions and higher-order structural dependencies.

Transformer architecture. A Transformer (5) consists of two main blocks: a self-attention module and a feed-forward neural network (FFN). In the self-attention module, Given input node features X, linear projections generate query ($\mathbf{Q}$), key ($\mathbf{K}$), and value ($\mathbf{V}$) matrices. The self-attention is then computed as:

$$Attn(X) = softmax(\frac{\mathbf{QK}^T}{\sqrt{d_q}})\mathbf{V} \in \mathbb{R}^{N\times d_q}. \tag{S1}$$

Where d_q represents the dimension of $\mathbf{Q}$, and $\mathbf{W_Q}$, $\mathbf{W_K}$, $\mathbf{W_V}$ are trainable parameters. We use multi-head attention, which concatenates multiple instances of Eq. (S1), a strategy shown to be effective in practice (5). As noticed by Mialon et al. (6), the self-attention in Eq. (S1) can be rewritten as a kernel smoother:

$$Attn(x_{v_i}) = \sum_{v_j\in V} \frac{k_{exp}(x_{v_i}, x_{v_j})}{\sum_{v_j\in V} k_{exp}(x_{v_i}, x_{v_j})} f(x_{v_j}), \forall v_i \in V. \tag{S2}$$

where $f(x) = \mathbf{W}_V x$ is the linear value function and k_{exp} is a (non-symmetric) exponential kernel on $\mathbb{R}^{d_q} \times \mathbb{R}^{d_q}$ parameterized by $\mathbf{W_Q}$ and $\mathbf{W_K}$:

$$k_{exp}(x, x^{'}) = exp(\frac{\langle \mathbf{W_Q} x, \mathbf{W_K} x^{'} \rangle}{\sqrt{d_q}}). \tag{S3}$$

where $\langle \cdot, \cdot \rangle$ is the dot product on $\mathbb{R}^{d_q}$. This formulation enables relative positional encoding through diffusion kernels, capturing positional similarity between nodes. However, it remains limited to position-awareness and does not explicitly model structural similarity.

Structure-aware self-attention.The main limitation of the kernel smoother is that it cannot distinguish structurally different nodes with similar features. To overcome this, we generalize the kernel to incorporate local subgraph information. By introducing a set of subgraphs centered at each node, the current structure-aware self-attention is defined as:

$$\mathrm{SA} - Attn(x_{v_i}) = \sum_{v_j \in V} \frac{k_{graph}(S_G(v_i), S_G(v_j))}{\sum_{v_j \in V} k_{graph}(S_G(v_i), S_G(v_j))} f(x_{v_j}). \tag{S4}$$

where $S_G(v_i)$ denotes a subgraph in G centered at a vertex v_i associated with vertex features X and k_{graph} can be any kernel that compares a pair of subgraphs (see Figure 2 in Main Text). This new self-attention function not only takes the attributed similarity into account but also the structural similarity between subgraphs. It thus generates more expressive node representations than the original self-attention. Moreover, this self-attention is no longer equivariant to any permutation of nodes but only to nodes whose features and subgraphs coincide. To facilitate the computation described above, we simplify the k_{graph} as follows:

$$k_{graph}(S_G(v_i), S_G(v_j)) = k_{exp}(\varphi(v_i, G), \varphi(v_j, G)). \tag{S5}$$

where $\varphi(v_i, G)$ is a structure extractor that extracts vector representations of some subgraph centered at v_i with vertex features X. The representation of the entire k-hop subgraph centered at v_i is directly computed using GNN, rather than being limited to the node representation v_i itself. Formally, when the k-hop neighborhood of node v_i (including itself) is denoted as $N_k(v_i)$, the node representation v_i is given by Eq. (S6), which has been strictly verified to pass the powerful 1-WL test (2).

$$\varphi(v_i, G) = \sum_{v_j \in N_k(v_i)} GNN_G^{(k)}(v_j). \tag{S6}$$

Final embedding pipeline.To jointly model attribute and structural similarities, we concatenate the subgraph embeddings with the original node features along the feature dimension. These combined embeddings are processed by a structure-aware self-attention layer, followed by residual connections with degree-based scaling, layer normalization, and a position-wise FFN (Figure 2 in Main Text). In addition, the degree factor is integrated into the skip connection to reduce the dominance of highly connected hubs (7):

$$x_{v_i}^{'} = x_{v_i} + 1/\sqrt{d_{v_i}} SA - attn(v_i). \tag{S7}$$

where d_{v_i} denotes the degree of vertex v_i. After a Transformer layer, the graph retains its original structure but updates node features to $G = (V, E, X^{'})$, where $X^{'} = \left\{x_1^{'}, x_2^{'}, ..., x_N^{'}\right\} \in \mathbb{R}^{N\times N}$ corresponds to the output of the Transformer layer. Finally, a global max pooling operation aggregates features into subject-level embeddings $H = \left\{H_f^1, H_f^2, ..., H_f^Z\right\} \in \mathbb{R}^{Z\times d_Z}$ for each subject corresponding to the Z atlases is obtained, where $H_f^Z \in \mathbb{R}^{1\times d_Z}$ denotes the feature vector obtained by SAT feature extraction from the Zth atlas.

1.3 Lightweight Attentional Feature Fusion Network

Specifically, LAFFNet applies learnable attention coefficients to each input feature and performs a weighted summation, with normalization enforced by a softmax layer to ensure stable fusion. By avoiding correlation-based weighting, LAFFNet prevents the dominance of highly correlated but uninformative features. Moreover, it requires only a single linear layer followed by softmax, thereby drastically reducing parameter overhead. From section 1.2, we obtained Z different features $H = \left\{H_f^1, H_f^2, ..., H_f^Z\right\} \in \mathbb{R}^{Z\times d_Z}$, sized as $d_1, d_2, ..., d_Z$ respectively for each subject derived from distinct atlases. As a versatile framework, LAFFNet can be easily generalized to other feature fusion models. To ensure dimensional consistency across different features, we first apply a feature transformation layer. This process converts the Z features into a unified d-dimensional representation, as defined in Eq. (S8).

$$\hat{H} = \sigma(Linear_{d_Z\times d}(H_f^1, H_f^2, ..., H_f^Z)) \in \mathbb{R}^{Z\times d}. \tag{S8}$$

where σ denotes a nonlinear activation function. In this work, we adopt tanh, which is particularly effective for computing cosine similarity compared with ReLU or sigmoid. $Linear_{d_Z\times d}$ denotes a fully connected layer with an input size of d_Z and an output size of d. Each input feature is transformed by a dedicated linear projection layer (with independent parameters), which can be omitted when the input dimension d_Z equals the output dimension d to preserve the original feature space. Although the features $\hat{H} = \left\{\hat{H}_f^1, \hat{H}_f^2, ..., \hat{H}_f^Z\right\} \in \mathbb{R}^{Z\times d}$ are now dimensionally consistent, their discriminative contributions remain unequal. To address this, we perform weighted fusion:

$$\bar{h} = \sum_{i=1}^{Z} a_i \hat{H}_i. \tag{S9}$$

where the attention weights $\{a_1, ..., a_Z\}$ are computed by a lightweight attention layer:

$$\{a_1, ..., a_Z\} = softmax(Linear_{d\times 1}(\hat{H}_f^1, \hat{H}_f^2, ..., \hat{H}_f^Z)) \in \mathbb{R}^Z. \tag{S10}$$

This process results in a d-dimensional fused vector representation ultimately. This formulation yields a convex combination of features, ensuring interpretability while maintaining computational efficiency (8), offering enhanced interpretability over MHSA.

The model's final output is achieved using a classifier for disease diagnosis. First, the fused feature vector $\bar{h}$ of each subject is weighted and summed according to formula (S9) to serve as input to the classifier. This vector has a dimension of $1 \times d$. Subsequently, this feature representation is processed through a multi-layer perceptron comprising: (i) two fully connected layers with nonlinear activation functions for feature transformation and (ii) a softmax layer for outputting category probabilities. The network is trained

using the cross-entropy loss:

$$\ell_{CE} = -\sum_{i=1}^{C} y_i log(F_y(\bar{h})). \tag{S11}$$

where y_i denotes ground-truth label, $F_y(\bar{h})$ represents the predicted probability distribution output by the model, and C is the total number of categories.

2 SUPPLEMENTARY RESULTS AND ANALYSIS

2.1 Hyperparameter Settings

To help researchers reproduce the experimental results of the comparison methods, Table S1 shows the optimal hyperparameter settings for these methods.

Table S1. Hyperparameter setting for comparison methods

Method	Hyperparameter				
	GNN layers	Batch size	Dropout	Learning rate	Epochs
SVM	-	-	-	-	100
GCN	3	16	0.3	0.001	100
BrainnetCNN	-	16	0.3	0.001	100
BrainGNN	2	8	0.3	0.001	100
MCRLN	4	4	0.3	0.1	800
MGCA-RAFFNet	3	16	0.5	0.001	500
SGCN	3	8	0.3	0.001	100
MHAHGEL	2	24	0.5	0.001	30
MSAT-LAFFNet (ours)	3	8	0.3	0.001	200

2.2 Ablation Study

First, the efficacy of the multi-atlas module was examined through extensive testing across different atlas combinations. Quantitative ablation studies revealed statistically significant differences ($p < 0.05$, FDR-corrected) between configurations. As shown in Tables S2 and S3, multi-atlas feature fusion (AAL116+CC200+BN246) consistently yielded significant performance gains compared with both single-atlas and dual-atlas settings. On the ABIDE-I dataset (Table S2), the tri-atlas configuration achieved minimum improvements of 3.46% in ACC and 3.18% in AUC relative to the best single-atlas (CC200) and dual-atlas approaches. Similarly, on the PTSD dataset (Table S3), the three-atlas combination provided at least 3.97% improvement in ACC and 4.71% in AUC compared with the best-performing single-atlas (BN246) and dual-atlas combinations. Several key insights emerged: (1) a more detailed atlas is not always beneficial, with 100-300 brain regions appearing most effective for ASD and PTSD characterization; (2) among single-atlas methods, the CC200 parcellation achieved superior results on the ABIDE-I dataset, while BN246 was optimal for PTSD classification; and (3) integrating more than four atlases led to nonlinear performance degradation, suggesting that excessive fusion introduces feature redundancy that hampers classification.

Second, the contributions of the SAT (structure-aware k-subgraph GNN extractor), AT (standard Transformer with GNN extractor without k-subgraph) and LAFF Modules were systematically evaluated using ACC(%), SEN(%), SPE(%) and AUC(%) distribution histograms (see Figure S1) under six conditions:

(i) removal of both the SAT and LAFF modules (baseline); (ii) AT only; (iii) SAT only; (iv) LAFF only; (v) inclusion of AT and LAFF; and (vi) inclusion of both the SAT and LAFF modules. Figure S1 demonstrates that both SAT and LAFF significantly enhance classification performance when combined with the multi-atlas strategy. Specifically, SAT module consistently outperformed the standard AT module. For instance, in ACC, SAT improved performance from the baseline (73.6% in PTSD task) to 81.3%, while AT achieved 78.2%. Compared to either graph Transformer variant (SAT or AT), the addition of LAFF further enhances performance. In ACC, LAFF reached 83.8% (PTSD) versus 81.3% for SAT and 78.2% for AT. In SEN, LAFF achieved 80.5% in the PTSD task versus 77.8% for SAT. In SPE, SAT showed advantages in certain configurations, with SAT LAFF achieving the highest specificity (86.2% in PTSD task). The same trend was also observed in ASD. The combination of SAT LAFF achieved the highest performance across all metrics and both classification tasks. In the PTSD task: ACC reached 89.4% (vs. 83.8% for LAFF alone), SEN reached 85.9%, SPE reached 86.2%, and AUC peaked at 90.0%. In the NC vs. ASD task: ACC reached 81.6%, SEN reached 80.3%, SPE reached 80.7%, and AUC reached 82.9%. The SATLAFF combination consistently outperformed ATLAFF across all metrics, confirming the superiority of the structure-aware mechanism. These results clearly demonstrate that the SAT module provides enhanced structural feature extraction, while the LAFF module enables effective feature fusion. Their combination yields synergistic improvements, with SAT LAFF achieving the optimal balance between sensitivity and specificity, leading to superior overall classification performance in both dateset.

Table S2. Performance comparison of ablation results for different atlas combinations in ASD dataset(%)

Atlas	ACC (%)	SEN(%)	SPE(%)	AUC(%)
AAL116	62.81±1.13	61.35±0.72	60.28±1.05	63.35±0.48
DOS160	62.29±1.31	61.51±1.22	60.84±1.03	63.34±0.57
CC200	72.27±0.69	71.22±0.81	71.55±0.63	74.17±0.66
BN246	72.11±0.45	71.08±1.34	71.01±1.08	73.67±0.54
Zalesky980	65.23±1.12	64.19±0.72	63.82±1.04	66.14±0.75
AAL116+CC200	77.86±1.36	76.43±1.01	76.04±1.06	79.19±0.53
**AAL116+CC200+BN246**	**81.08±0.51**	**79.23±1.03**	**80.31±0.42**	**82.23±0.67**
AAL116+DOS160+CC200+BN246	69.24±1.31	68.42±1.27	67.55±4.02	70.73±1.41

Results are expressed as “Mean ± standard deviation.” Bold fonts indicate the best performance.

Table S3. Performance comparison of ablation results for different atlas combinations in PTSD dataset(%)

Atlas	ACC(%)	SEN(%)	SPE(%)	AUC(%)
AAL116	72.81±1.09	76.35±0.65	67.87±1.18	70.35±0.65
DOS160	72.36±1.28	75.29±0.71	72.41±1.36	74.29±0.71
CC200	77.22±0.71	79.16±0.73	73.67±0.64	75.16±0.73
BN246	77.91±0.56	82.05±1.21	79.1±1.58	78.67±0.33
Zalesky980	70.22±1.43	73.16±0.67	83.12±1.82	82.68±1.31
AAL116+CC200	83.95±1.53	84.08±1.31	84.52±1.25	85.12±0.13
**AAL116+CC200+BN246**	**88.26±1.19**	**89.51±0.45**	**84.62±1.57**	**89.51±0.45**
AAL116+DOS160+CC200+BN246	70.15±1.17	72.64±1.58	82.08±1.02	77.12±2.92

Results are expressed as “Mean ± standard deviation.” Bold fonts indicate the best performance.

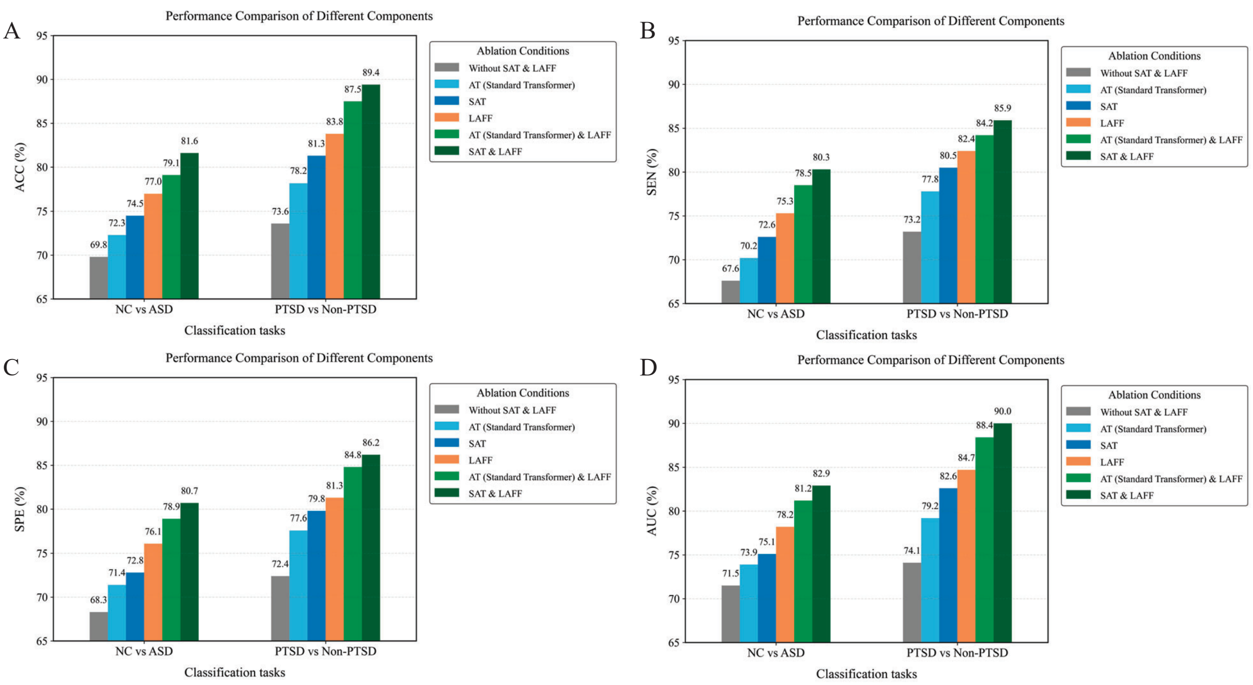

Figure S1. The Impact of the SAT, AT, and LAFF modules on evaluation metrics across classification tasks: **(A)** ACC, **(B)** SEN, **(C)** SPE, **(D)** AUC.

2.3 Computational Complexity

In this section, we provide an in-depth evaluation of the computational complexity and efficiency of the proposed model. Table S4 reports the number of trainable parameters and floating-point operations (FLOPs). Compared with the MHSA mechanism, the proposed LAFF module exhibits superior data efficiency owing to its reduced parameter count. Moreover, LAFF offers enhanced interpretability, as its attention weights are directly utilized to construct convex combinations of feature representations. This enables a more transparent correspondence between learned parameters and feature transformations, thereby improving both computational efficiency and model explainability.

Table S4. Complexity analysis of feature fusion blocks. D and d represent the input and output feature dimensions, respectively. FLOPs are computed for input features of shape 8×2080.

Feature fusion block	Parameters	FLOPs(M)
MHSA	$D \times d + 4 \times d^2$	49.94
LAFF	$D \times d + d$	13.48

2.4 Impact of the Hyperparameters of SAT

Two critical hyperparameters of the proposed model are the subgraph neighborhood size k (k-hop neighbor) and the number of attention heads h. The parameter k governs the distribution of subgraph attention weights, while h balances the trade-off between expressiveness and computational efficiency of the multi-head attention mechanism. To investigate their impact on classification performance, we systematically varied k from 1 to 4 (step = 1) and h from 1 to 10 (step = 1). The corresponding ACC results

are illustrated in Figure S2. The findings indicate that the optimal setting of k=2 and h=8 achieves the highest classification accuracy for both NC vs. ASD and PTSD vs. Non-PTSD tasks.

Furthermore, the results reveal that excessively small or large values of k and h lead to degraded performance. Specifically, a large k introduces noise and increases computational and memory overhead, whereas a small k restricts the model to local features from immediate neighbors, thereby reducing discriminative power. Similarly, a small h leads to underfitting, while a large h substantially increases computational cost and the risk of overfitting.

In addition to k and h, other hyperparameters of the proposed model, including learning rate, batch size, and maximum training epochs, were systematically optimized using a grid search strategy. This procedure employed a Cartesian product-based exploration of predefined parameter spaces, ensuring a thorough assessment of critical configurations while maintaining reproducibility (9).

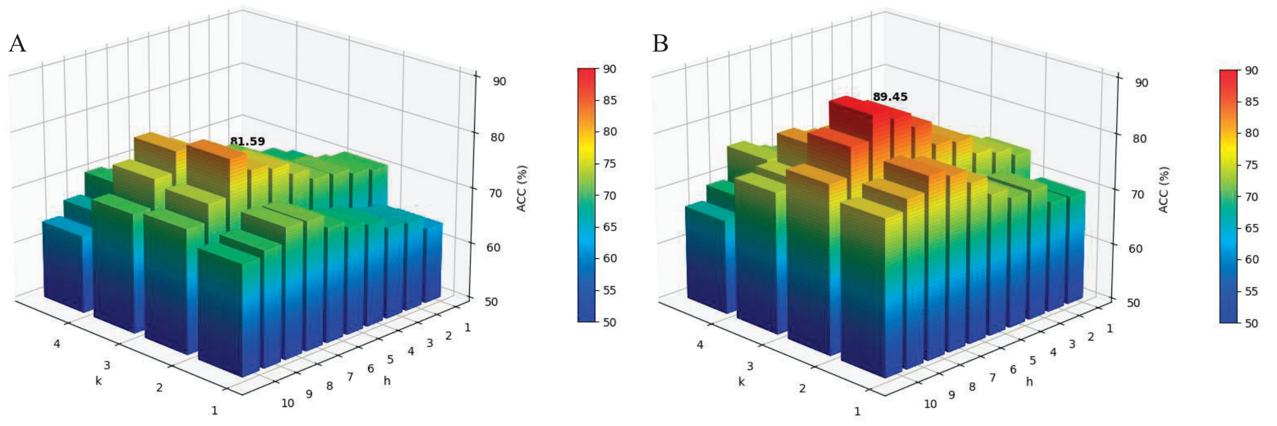

Figure S2. The influence of the hyper-parameters k and h on classification performance. **(A)** NC vs. ASD, **(B)** Non-PTSD vs. PTSD.

REFERENCES

1. Wu Z, Pan S, Chen F, Long G, Zhang C, Yu P S . A comprehensive survey on graph neural networks. *IEEE Trans Neural Netw Learn Syst.* (2020),32(1): 4–24. doi:10.1109/TNNLS.2020.2978386.

2. Kreuzer D, Beaini D, Hamilton W, Létourneau V, Tossou P. Rethinking graph Transformers with spectral attention. presented at the 35th Conf Neur Inf Proc Syst. NeurIPS, Montreal, Canada, Sep. 21618-21629, 2021.

3. Dwivedi V P, Luu A T, Laurent T, Bengio Y, Bresson X. Graph neural networks with learnable structural and positional representations. (2021) arXiv:1609.02907.

4. Chen D, O'Bray L, Borgwardt K. Structure-aware Transformer for graph representation learning. presented at the 39th Int Conf Mach Learning. PMLR, Maryland, USA, Jul. 3469-3489, 2022.

5. Vaswani A, Shazeer N, Parmar N, Uszkoreit J, Jones L, Gomez A N, et al. Attention is all you need. presented at the 31th Conf Neur Inf Proc Syst. NeurIPS, Long Beach, USA, Sep. 1-11, 2017.

6. Mialon G, Chen D, Selosse M, Mairal J. Graphit: Encoding graph structure in Transformers. (2021) arxiv:2106.05667.

7. Zhang M, Li P. Nested graph neural networks. presented at the 35th Conf Neur Inf Proc Syst. NeurIPS, Sep. 15734-15747, 2021.

8. Hu F, Chen A, Wang Z, Zhou F, Dong J, Li X. Lightweight attentional feature fusion: A new baseline for text-to-video retrieval. presented at the Euro. Conf. Comp Vision. ECCV, Switzerland, Oct. 444-461, 2022.

9. Ma Y, Cui W, Liu J, Guo Y, Chen H, Li Y. A multi-graph cross-attention-based region-aware feature fusion network using multi-template for brain disorder diagnosis. *IEEE Trans Med Imag.* (2023),43(3): 1045–1059. doi:10.1109/TMI.2023.3327283.
